# Thermogenic and hemodynamic effects of ingesting a pre-workout supplement with and without synephrine

**DOI:** 10.1186/1550-2783-11-S1-P35

**Published:** 2014-12-01

**Authors:** YP Jung, C Goodenough, M Cho, A O’Connor, R Dalton, K Levers, E Galvan, N Barringer, F Ayadi, J Carter, M Koozechian, S Simbo, A Reyes, B Sanchez, A Coletta, C Rasmussen, R Kreider

**Affiliations:** 1 Texas A&M University, College Station, Texas, USA

## Background

A number of nutritional strategies have been developed to optimize nutrient delivery prior to exercise. As a result, a number of pre-workout supplements have been developed to increase energy availability, promote vasodilation, and/or positively affect exercise capacity. The purpose of this study was to examine the acute effects of ingesting a pre-workout dietary supplement with and without synephrine on energy metabolism and cardiovascular hemodynamics.

## Methods

In a double-blind, crossover, randomized and placebo-controlled manner; 25 apparently healthy and recreationally active men and women (21.76±3.00 yr, 15.24±5.26% fat, 25.09±3.03 kg/m2) volunteered to participate in this study and had resting blood pressure (BP), heart rate (HR), 12-lead electrocardiographs (ECG), and resting energy expenditure (REE) measured for 10 minutes. Participants then ingested in a randomized and counterbalanced manner a dextrose flavored placebo (P); a pre-workout supplement (PWS) containing 3.0 g beta alanine, 2 g creatine nitrate, 2 g arginine AKG, 300 mg of N-acetyl tyrosine, 270 mg caffeine, 15 mg of Mucuna pruriens; or, the PWS with 20 mg of synephrine (PWS+S). Metabolic changes were measured continuously while BP, HR, and ECG’s were obtained every 10 minutes during the REE test. Participants repeated the experiment after a one week washout period with the alternate supplements in a randomized and counterbalanced manner. Data were analyzed by repeated measure MANOVA and are presented as means ± SD or SEM from baseline. Consent to publish the results was obtained from all participants.

## Results

MANOVA analysis revealed a significant overall Wilks’ Lambda time (p<0.001) and time x group interactions (p<0.001) for oxygen uptake (VO2), carbon dioxide production (VCO2), minute ventilation (Ve), respiratory exchange ratio (RER), and REE values. MANOVA Greenhouse-Geisser univariate analysis revealed significant interactions among groups in VCO2 (p=0.003) and RER (p<0.001) with a trend toward significance in REE (p=0.098). Delta analysis revealed significant differences among groups in mean change in VO2 (P: 3.8±5.2; PWS: 15.4±5.2; PWS+S: 23.5±5.2 ml/min; p=0.03), VCO2 (P: 12.5±5.1; PWS: 31.8±5.1; PWS+S: 37.7±5.1 ml/min; p=0.002), RER (P: 0.033±0.009; PWS: 071±0.009; PWS+S: 0.071±0.009; p=0.005), and REE (P: 0.034±0.025; PWS: 0.095±0.025; PWS+S: 0.132±0.025 kcal/min; p=0.02) with significant differences observed among the P group and both supplemented groups. PWS-S ingestion promoted a more prominent increase in VO2, VCO2, and REE during the initial 5-10 minutes after ingestion with differences minimizing thereafter. Area under the curve (AUC) analysis of changes from baseline revealed that PWS+S and PWS supplementation resulted in significantly greater AUC values than P in VO2 (PWS+S: 1,034±584; PWS: 802±434; P: 684±376; p=0.01); VCO2 (PWS+S: 1,372±604; PWS: 1,151±604; P: 634±262; p<0.01); and RER (PWS+S: 2.79±0.89; PWS: 2.44±0.98; P: 1.46±0.66; p<0.01). There were no significant interaction effects for HR (p=0.77), SBP (p=0.35), or DBP (p=0.65) and there was no evidence of an increase in ECG assessed arrhythmias during the REE assessment.

## Conclusion

Ingesting a PWS containing beta alanine, creatine nitrate, arginine AKG, N-Acetyl Tyrosine, caffeine, and Mucuna pruriens increased resting VO2, VCO2, RER, and tended to increase REE values in comparison to a placebo. Addition of 20 mg of synephrine to the PWS resulted in a greater increase in the metabolic response during the first 5-10 minutes after ingestion but differences were not as apparent thereafter and AUC values were not significantly different between the PWS and PWS+S groups. PWS and PWS+S ingestion did not result in a significantly different HR or BP responses during the REE test in comparison to P responses. Results indicate that ingestion of these pre-workout supplements promoted modest thermogenic response and that addition of 20 mg of synephrine to the PWC provided limited additional benefit.

